# A collaborative approach to adopting/adapting guidelines - The Australian 24-Hour Movement Guidelines for the early years (Birth to 5 years): an integration of physical activity, sedentary behavior, and sleep

**DOI:** 10.1186/s12889-017-4867-6

**Published:** 2017-11-20

**Authors:** Anthony D. Okely, Davina Ghersi, Kylie D. Hesketh, Rute Santos, Sarah P. Loughran, Dylan P. Cliff, Trevor Shilton, David Grant, Rachel A. Jones, Rebecca M. Stanley, Julie Sherring, Trina Hinkley, Stewart G. Trost, Clare McHugh, Simon Eckermann, Karen Thorpe, Karen Waters, Timothy S. Olds, Tracy Mackey, Rhonda Livingstone, Hayley Christian, Harriette Carr, Adam Verrender, João R. Pereira, Zhiguang Zhang, Katherine L. Downing, Mark S. Tremblay

**Affiliations:** 1 Early Start, Faculty of Social Sciences, University of Wollongong, Wollongong, NSW 2522 Australia; 2 Illawarra Health and Medical Research Institute, Wollongong, Australia; 3 Research Policy and Translation, National Health and Medical Research Council, Canberra, Australia; 4 National Health & Medical Research Council Clinical Trials Centre, Sydney Medical School, University of Sydney, Sydney, Australia; 5 Institute for Physical Activity and Nutrition, School of Exercise and Nutrition Sciences, Deakin University, Geelong, Australia; 6 Faculty of Sport, University of Porto, Porto, Portugal; 7 School of Psychology, Faculty of Social Sciences, University of Wollongong, Wollongong, Australia; 8 National Heart Foundation (WA), 334 Rokeby Road, Subiaco, Australia; 9 Population Health and Sport Division, Australian Government Department of Health, Canberra, Australia; 10 Institute of Health and Biomedical Innovation at Queensland Centre for Children’s Health Research, Queensland University of Technology, Brisbane, Australia; 11 Early Childhood Australia, Canberra, Australia; 12 Australian Health Services Research Institute, University of Wollongong, Wollongong, Australia; 13 Institute for Social Science Research, The University of Queensland, Brisbane, Australia; 14 Children’s Hospital Westmead and University of Sydney, Sydney, Australia; 15 Alliance for Research in Exercise Nutrition and Activity (ARENA), Sansom Institute, School of Health Sciences, University of South Australia, Adelaide, Australia; 16 NSW Department of Education, Sydney, Australia; 17 Australian Children’s Education & Care Quality Authority (ACECQA), Sydney, Australia; 18 School of Population and Global Health and Telethon Kids Institute, The University of Western Australia, Perth, Australia; 19 New Zealand Ministry of Health, Wellington, New Zealand; 20 Healthy Active Living and Obesity Research Group, Children’s Hospital of Eastern Ontario Research Institute, Ottawa, Canada

**Keywords:** Methodology, Infants, Toddlers, Preschoolers, GRADE-ADOLOPMENT, Public health recommendations

## Abstract

**Background:**

In 2017, the Australian Government funded the update of the National Physical Activity Recommendations for Children 0–5 years, with the intention that they be an integration of movement behaviours across the 24-h period. The benefit for Australia was that it could leverage research in Canada in the development of their 24-h guidelines for the early years. Concurrently, the Grading of Recommendations Assessment, Development and Evaluation (GRADE) working group published a model to produce guidelines based on adoption, adaption and/or de novo development using the GRADE evidence-to-decision framework. Referred to as the GRADE-ADOLOPMENT approach, it allows guideline developers to follow a structured and transparent process in a more efficient manner, potentially avoiding the need to unnecessarily repeat costly tasks such as conducting systematic reviews. The purpose of this paper is to outline the process and outcomes for adapting the *Canadian 24-Hour Movement Guidelines for the Early Years* to develop the *Australian 24-Hour Movement Guidelines for the Early Years guided by the GRADE-ADOLOPMENT framework.*

**Methods:**

The development process was guided by the GRADE-ADOLOPMENT approach. A Leadership Group and Consensus Panel were formed and existing credible guidelines identified. The draft Canadian 24-h integrated movement guidelines for the early years best met the criteria established by the Panel. These were evaluated based on the evidence in the GRADE tables, summaries of findings tables and draft recommendations from the Canadian Draft Guidelines. Updates to each of the Canadian systematic reviews were conducted and the Consensus Panel reviewed the evidence for each behaviour separately and made a decision to adopt or adapt the Canadian recommendations for each behaviour or create de novo recommendations. An online survey was then conducted (*n* = 302) along with five focus groups (*n* = 30) and five key informant interviews (*n* = 5) to obtain feedback from stakeholders on the draft guidelines.

**Results:**

Based on the evidence from the Canadian systematic reviews and the updated systematic reviews in Australia, the Consensus Panel agreed to adopt the Canadian recommendations and, apart from some minor changes to the wording of good practice statements, keep the wording of the guidelines, preamble and title of the Canadian Guidelines. The Australian Guidelines provide evidence-informed recommendations for a healthy day (24-h), integrating physical activity, sedentary behaviour (including limits to screen time), and sleep for infants (<1 year), toddlers (1–2 years) and preschoolers (3–5 years).

**Conclusions:**

To our knowledge, this is only the second time the GRADE-ADOLOPMENT approach has been used. Following this approach, the judgments of the Australian Consensus Panel did not differ sufficiently to change the directions and strength of the recommendations and as such, the Canadian recommendations were adopted with very minor alterations. This allowed the Guidelines to be developed much faster and at lower cost. As such, we would recommend the GRADE-ADOLOPMENT approach, especially if a credible set of guidelines, with all supporting materials and developed using a transparent process, is available. Other countries may consider using this approach when developing and/or revising national movement guidelines.

## Background

In 2008, the Australian Government funded the development of the first national recommendations for physical activity and sedentary behaviour in the early years (defined as ages 0–5 years and not yet attending school). Released in 2010 [1], they were followed by national guidelines in the United Kingdom [2] and Canada [3, 4]. More recently, there has been a move to develop guidelines that take into account, from a movement perspective, the entire day. Referred to as 24-h integrated movement guidelines [5] they acknowledge that the whole day matters and individual movement behaviours such as physical activity, sedentary behaviour and sleep need to be considered in relation to each other when examining their associations with health and developmental outcomes in children. In 2016, Canada was the first country to release integrated 24-h movement guidelines for school-aged children and youth [5]. These guidelines reinforce the importance of considering the integration of movement behaviours with evidence showing a monotonic relationship between the number of movement behaviour guidelines met by an individual and associated health indicators [6–8]. That is, meeting all three guidelines was better than meeting any two, and meeting any combination of two guidelines was better than meeting just one.

In November 2015, Canada commenced the process of updating their early years guidelines to reflect 24-h integrated movement guidelines. This process, which followed the GRADE framework for guideline development [9], culminated with Canada’s first 24-h movement guidelines for this age group which are part of this issue [10]. In early 2017, the Australian Government provided funding to update the National Physical Activity Recommendations for Children 0–5 years, to be an integration of movement behaviours across the 24-h period, consistent with the Canadian guidelines. The potential benefit for Australia was that it could leverage the considerable work done in Canada in the development of their 24-h guidelines to complete what would normally be a much longer process, in considerably less time and requiring fewer resources. The purported benefits of adapting guidelines produced by others had not been demonstrated and one of the aims of this paper was to demonstrate the successful adaptation of one national guideline into another [10].

The GRADE-ADOLOPMENT approach allows guideline developers to follow a well-accepted and transparent process for developing guidelines (GRADE) in an efficient manner by adapting or adopting an existing evidence-based guideline. This could potentially prevent the need to undertake (or repeat) costly tasks such as conducting full systematic reviews [11]. At the same time, it allows local guideline developers to take into consideration factors that are specific to their local context.

Based on the Canadian Guideline Development Panel’s use of the GRADE approach to develop the *Canadian 24-Hour Movement Guidelines for the Early Years* (some 12–18 months earlier), it was decided to use the GRADE-ADOLOPMENT approach in the development of the *Australian 24-Hour Movement Guidelines for the Early Years*. The purpose of this paper was to outline the process and outcomes for the ADOLOPMENT of the *Canadian 24-Hour Movement Guidelines for the Early Years* to develop the *Australian 24-Hour Movement Guidelines for the Early Years*. This process started in February 2017 and was completed by the end of June 2017, with Guideline release coordinated to occur concurrently with Canada in November 2017.

## Methods

### Guideline ADOLOPMENT structure

**Fig. 1:**
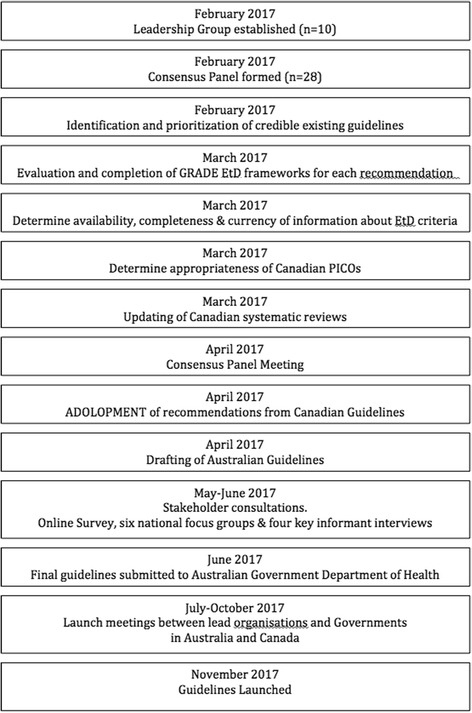
Timeline and sequence of steps involved in the development of the Australian 24-Hour Movement Guidelines for the Early Years (birth to 5 years): An Integration of Physical Activity, Sedentary Behaviour, and Sleep. (refs: [11, 24]). Abbreviations: EtD: evidence-to-decision frameworks; GRADE: Grading of Recommendations Assessment, Development, and Evaluation; PICO: Population, Intervention, Comparator, and Outcome

The GRADE-ADOLOPMENT process followed the framework described in detail by Schünemann et al. 2017 [11]. In addition, several steps that were identified in the Appraisal of Guidelines for Research & Evaluation II (AGREE-II) instrument were included [12]. A summary of the timeline and sequence of steps we used is shown in Fig. 1.

*Step 1: Establishment of a Leadership Group.* This group comprised the project Principal Investigators (ADO, KDH, RS, DPC, SPL, MST) and representatives from the Australian Government (owner and funder of the Guidelines; DGr), National Heart Foundation of Australia (key stakeholder; TS), and professional support from Early Start at the University of Wollongong (JS). This group was formed in February 2017 and met fortnightly up to the end of June 2017 to provide strategic advice and direction, guidance, and budget accountability to the project. A guideline methodologist was invited to be part of the leadership group (DGh) with all members making a commitment to using the GRADE-ADOLOPMENT approach. Ad-hoc subcommittees were formed around the areas of stakeholder consultation (RMS, RAJ), dissemination and implementation (SE, RAJ, ADO), and surveillance (ADO, KDH, RS, DPC, SPL, SGT, TH, TSO) at appropriate time points in the process. As the Australian guidelines sought to adopt or adapt the Canadian Guidelines using the GRADE-ADOLOPMENT process (assuming these would be appropriate as per Step 3 – see below for details), it was agreed that the Principal Investigator from the Canadian Guidelines (MST) and the Principal Investigator from the Australian Guidelines (ADO) would be part of each other’s country leadership group to ensure communication and collaboration across countries. This was particularly important as Canada had not yet completed their guideline development process (their second consensus meeting occurred in January 2017) and it was critical that the Australian team were aware of how the Canadian process was progressing, especially in light of any changes that were made. This was necessary as both countries were working towards a co-release of the guidelines.

*Step 2: Formation of a Consensus Panel.* A guideline development Consensus Panel was also formed which included expert researchers, representatives from key stakeholder groups (including parents and Indigenous communities), and methodology experts (Table 1). The role of this Panel is described in detail in Step 5. Efforts were made to achieve geographical representation across Australia within the confines of the budget.

**Table 1:** Guideline Consensus Panel

Panel Member	Affiliation	Role	Conflict of Interest Declaration
Research Experts			
Kylie Hesketh	Deakin University, Melbourne, Australia	Researcher, expert PA	Receive funding from ARC, NHMRC and Heart Foundation. Serves on the Steering Committee of Parents Voice. Member of the Active Healthy Kids Australia Executive Committee that produces the Physical Activity Report Card. On the Editorial Board of the International Journal of Behavioural Nutrition and Physical Activity. Has published journal articles on children’s physical activity and sedentary behaviour. Has given presentations on children’s physical activity and sedentary behaviour. Has been involved in writing reports which include content on children’s physical activity and sedentary behaviour for WHO, Heart Foundation, state and federal governments.
Rute Santos	University of Wollongong, Wollongong, Australia	Researcher, expert SB	I have a Discovery Early Career Research Award from the Australian Research Council. I am a member of International Behaviour Research Network I am a member of the NCDS Risk Factor Collaboration group.
Sarah Loughran	University of Wollongong, Wollongong, Australia	Researcher, expert Sleep	Current funding and subsequent publications on screen time, mobile phones and sleep in children (NHMRC), including two opinion pieces in The Conversation on screen time and sleep. A member of the WHO environmental Health Criterion RF expert group.
Dylan Cliff	University of Wollongong, Wollongong, Australia	Researcher, expert SB, PA, compositional analyses	Has received funding from ARC and NHMRC Has published journal articles and given presentations on physical activity, sedentary behaviour and electronic media use in children. Consultancy to Early Childhood Australia to deliver Munch & Move Professional Development for early childhood educators in NSW.
Stewart Trost	Queensland University of Technology, Brisbane, Australia	Researcher, expert PA, SB	Received funding from NIH, ARC, and NHMRC. Member of the Actigraph Corporation Scientific Advisory Board
Hayley Christian	University of Western Australia, Australia	Researcher, expert PA, SB	Current research funding from Healthway for related projects. National Heart Foundation Future Leader Fellowship to conduct related research Publications and presentations in this area Funding (travel and accommodation) to attend this meeting International Society for Behavioural Nutrition and Physical Activity “Child related” SIGS Confidential Information – study participants as per research area and peer review
Anthony Okely	University of Wollongong, Wollongong, Australia	Chair, researcher, content expert PA, SB	Have received funding from NHMRC and ARC for related projects Member of Consensus Committee for Canadian 24-h integrated movement guidelines for the early years (travel and accommodation covered) Member of Consensus Committee for Canadian 24-h integrated movement guidelines for children and youth (travel and accommodation covered) Paid consultancy from Foxtel on active interstitials for children’s pay television channels. Consultancy to Early Childhood Australia to deliver Munch & Move Professional Development for early childhood educators in NSW.
Rachel Jones	University of Wollongong, Wollongong, Australia	Researcher, expert knowledge translation	Consultancy to Early Childhood Australia to deliver Munch & Move Professional Development for early childhood educators in NSW. Has received grants from University of Wollongong, NHMRC. Has published journal articles on early childhood physical activity and sedentary behaviours, factors associated with physical activity in early childhood, outcomes of physical activity early childhood interventions. Has spoken at conferences/provided speeches and lectures on topics such as those in published journal articles
Trina Hinkley	Deakin University, Melbourne, Australia	Researcher, expert SB, PA	Funded by NHMRC ECF: PA/SB in early childhood. Pending ARC DECRA focusing on screen time in early childhood Secretary International Society of Behavioural Nutrition and Physical Activity and Member Early Care and Education SIG (previously co-chair). Has received research grants from Deakin University, Universities Australia: German Academic Exchange Service, National Research Foundation of South Africa Competitive Programme for Rated Researchers Has published journal articles on early childhood physical activity and sedentary behaviours, children’s compliance with existing recommendations and associations of the behaviours with cognitive development and psychosocial wellbeing Has spoken at conferences/provided speeches and lectures on topics such as those in published journal articles Has developed material related to the topic for various intervention programs
Tim Olds	University of South Australia, Adelaide, Australia	Expert sleep, compositional analyses	Employment 0.4 Research Professor University of South Australia Member of Consensus Committee for Canadian 24-h integrated movement guidelines for children and youth (travel and accommodation covered) NHMRC Project Grant Support ARC Support
Karen Thorpe	University of Queensland, Brisbane, Queensland (formerly at Queensland University of Technology, Brisbane, Australia)	Researcher, expert in sleep	Employment: University of Queensland Adjunct positions: University of Melbourne, University of Queensland Research Funding: Department of Education and Training, Queensland, Australian Research Council, National Health and Medical Research Council; Research Interests: Sleep in Early Childhood; Development of Sleep Professional Development for Exec Educators Publications: Early Childhood Education and Care, Sleep
Rebecca Stanley	University of Wollongong, Wollongong, Australia	Researcher, expert stakeholder consultation,	Funded by NSW Health Early-Mid Career fellow (from 25 May 2017). Membership of the International Society of Behavioural Nutrition and Physical Activity, Children and Families Special Interest Group, Sedentary Behaviour Research Network, NSW Cardiovascular Research Network for Early Career and Mid-Career Researchers Research interest for NSW Health fellowship is Indigenous Health which will result in publications and conference presentations. Funding: University of Wollongong Global Challenges Project Grant for the development, implementation and evaluation of afterschool cultural Employment: Project Manager of an NHMRC funded project grant on a randomized controlled trial in preschoolers focusing on physical activity and gross motor development, which will result in publications and conference presentations.
Katherine Downing	Deakin University, Melbourne, Australia	PhD student for Systematic review of Physical Activity	Funded through an NHMRC Postgraduate Scholarship. Has published journal articles on children’s sedentary behaviour. Has given presentations on children’s sedentary behaviour
Zhiguang Zhang	University of Wollongong, Wollongong, Australia	PhD student for Systematic review Integrated Movement Behaviours	Funded through an PhD scholarship from the China Scholarship Council
Joao Pereira	University of Wollongong, Wollongong, Australia	PhD student for Systematic review on Sedentary Behaviour	Funded through an UOW University Postgraduate Award Postgraduate scholarship
Adam Verrender	University of Wollongong, Wollongong, Australia	PhD student for Systematic review on Sleep	Funded through a joint NHMRC and UOW Postgraduate Scholarship
Stakeholder Group and Knowledge Users			
David Grant	Commonwealth Department of Health, Canberra, Australia	Stakeholder, end user	
Trevor Shilton	National Heart Foundation of Australia, Perth, Australia	Stakeholder, cardiovascular health, messaging	Member of the Board, International Society of Physical Activity and HealthMember of the Board International Union for Health Promotion and Education.
Tracy Mackey	Executive Director, Early Childhood, NSW Dept of Education	Stakeholder	Nil
Rhonda Livingstone	Australian Children’s Education & Care Quality Authority (ACECQA)	Stakeholder	A member of the Executive Team (and National Education Leader) of ACECQA
Karen Waters	Children’s Hospital Westmead and University of Sydney	Paediatrician, expert Sleep	Nil
Alice Pryor	Parents Voice	Parent advocate	
Clare McHugh	Early Childhood Australia, Canberra, Australia	Stakeholder	As part of my work with Early Childhood Australia, I manage the Digital Business Kit grant (Commonwealth ending June 2017) am an investigator on Smart Start (Research Coordinator) and coordinate online resources to support good pedagogical practices with technology, educators and young children. ECA is part of a number of grant applications relating to digital technology and good practices. ECA is working with a digital policy group to consult and develop a Guidance framework for the early childhood education and care of young children and digital technology use. ECA is planning to develop *Live Wires* an online platform (magazines, forums) to advise, inform and provide expert reviews on technology, products and tools. We will develop protocols for managing this Cubetto (Primo Toys) was provided to ECA (for review) by the manufacturer. ECA runs KidsMatter Early Childhood Wellbeing and Mental Health programs funded by the Commonwealth Government.
International Collaborators			
Mark Tremblay	Children’s Hospital of Eastern Ontario Research Institute, Ottawa, Canada	Chair of Canadian Guideline Panel, researcher, content expert PA, SB.	I have no financial interests but I am involved in the Canadian 24 Hour Guidelines for the Early Years (0–4 years). An integration of Physical Activity, Sedentary Behaviour and Sleep – and this involvement may be perceived as a conflict of interest. My expenses to attend the Australian Guideline Development meeting were covered but I received no honorarium. I donated my time for all aspects of my involvement.
Harriette Carr	New Zealand Ministry of Health, Wellington, New Zealand	Stakeholder, international	During the period of development of the Australian Guidelines, New Zealand were finalising their new Sit Less, Move More, Sleep Well: Active Play Guidelines for under Fives (released May 2017). Due to the close relationship between Australia and New Zealand, we wanted to ensure that our respective Guidelines were broadly consistent.
Methodology Consultants and Project Management			
Davina Ghersi	NHMRC (Canberra), Australia	GRADE-ADOLPMENT, AGREE methodology expert	I am an employee of NHMRC, an agency that approves and produces Guidelines. I also provide advice to WHO, in relation to Guidelines, specifically in nutrition.
Simon Eckermann	University of Wollongong, Wollongong, Australia	Health Economist	I have no financial interests. I have developed methods that may be used as part of evaluation, that are completed in the text Health Economics from Theory to Practice (Eckermann, 2017)
Julie Sherring	University of Wollongong, Wollongong, Australia	Project management	I am Project Manager for the development of the Australian 24-h Movement Guidelines for Children of the Early Years.

*ACECQA* Australian Children’s Education & Care Quality Authority, *ARC* Australian Research Council, *ECA* Early Childhood Australia, *NHMRC* National Health & Medical Research Council, *NSW* New South Wales, *UOW* University of Wollongong

*Step 3: Identification of credible existing guidelines and definition of criteria for selection of the guidelines.* We were aware of two sets of 24-h integrated movement guidelines for the early years. These were from Canada and New Zealand and, at the time (February 2017), were identified as being in development. The Canadian integrated Guidelines (then in development) were considered along with other existing integrated or physical activity and sedentary behaviour guidelines that met the following criteria: 1) published in the past 5 years (or in the process of being published); 2) addressed clear research questions (contained all Population, Intervention, Comparator and Outcome [PICO] elements); 3) followed the GRADE process; 4) allowed for updating (provided access to full systematic reviews, which were registered with the Prospective Register of Systematic Reviews (PROSPERO) and provided full access to the search strategy); 5) existing and accessible GRADE tables and summaries of findings; and 6) completed a risk-of bias assessment [11]. Table 2 contains a summary of the five sets of national physical activity and sedentary behaviour guidelines in the early years that the leadership group was able to find and the evaluation of these against these criteria. Using our existing networks we were unable to identify any other 24-h guidelines for the early years. Only the 2017 Canadian 24-Hour Movement Guidelines for the Early Years met these criteria and were therefore chosen as the guidelines to be adopted or adapted following the GRADE-ADOLOPMENT process.

**Table 2:** Existing Early Years Guidelines

Criteria	Australia 2010 [1]	UK 2011 [2]	Canada 2012 [3, 4]	New Zealand 2017^a^ [42]	Canada 2017^a^ [10]
Published in last 5y	N	N	Y	Y	Y
Followed GRADE process	N	N	Y	N	Y
Addressed clear questions (can identify PICO elements)	?	N	Y	?	Y
Had benefits and harms assessments	?	?	Y	?	Y
Assessed using AGREE	N	N	Y	N	Y
Allowed for updating	?	N	Y	?	Y
Had existing and accessible evidence tables/summaries	?	N	Y	N	Y
Had risk of bias assessment	N	N	Y	N	Y
Were integrated (24 h)	N	N	N	Y	Y

Reference: Appendix 1. GRADE-ADOLOPMENT [11]

*Y* yes, *N* no, *?* unsure

^a^under development during guideline development process but made available to Australian Consensus Panel

The AGREE-II tool was used to determine the credibility of the 2017 Canadian Guidelines (as per Stage 1 of the suggested GRADE-ADOLOPMENT Protocol – see Appendix 1 [11]). As the AGREE tool was developed to enable end users to assess the quality of a completed guideline, some of the items are not relevant to guidelines in development. This includes AGREE Domains 4 (clarity of presentation) and 5 (applicability). Although the Canadian guidelines were not yet published at the time the Australian Guidelines were being developed, and the AGREE-II form could not be completed in its entirety, we were confident based on the nature of our relationship that the ratings for each of the AGREE-II criteria would be high. Following the credibility assessment the ADOLOPMENT framework moves on to the evaluation and final selection of the guidelines that will be adopted or adapted. It was agreed by the Leadership Group that it would be appropriate to adopt the Canadian Guidelines as they were determined to be of appropriate quality, their scope/applicability was appropriate for Australia, the topic was a priority for Australia and the research questions and PICOs (Population, Intervention, Comparators, and Outcomes) for the systematic reviews that served as the evidence base were relevant.

*Step 4: Evaluate and complete GRADE Evidence-to-Decision (EtD) frameworks for each recommendation.* The Australian Consensus Panel considered the evidence-to-decision criteria that influenced the direction and strength of each of the draft recommendations made by the Canadian Guideline Development Panel based on the GRADE tables, summary of findings tables, and draft recommendations made available by the 2017 Canadian Guideline Leadership Committee.

In most cases, assessed against the stated GRADE approach to evidence synthesis (60% of RCT studies statistically significant and positive), the evidence base was graded “Low” or “Very Low”. The Consensus Panel then made a decision to support the draft 2017 Canadian Guidelines or not based on the evidence and other criteria used to make recommendations including values and preferences; feasibility, acceptability and equity issues; resources; balance of benefits and harms; and quality of the evidence [12]. Parts of the EtD framework able to be followed during the Consensus Panel meeting included presenting the evidence and keeping track of the discussion and judgments. Following the Consensus Panel meeting, a transparent record of the discussions was communicated to those who attended.

*Step 5 Determine availability, completeness & currency of information about EtD criteria.* The next component in the general stages of GRADE-ADOLOPMENT (see Appendix 3 [11]) was to determine the availability, completeness, and currency of the information about the EtD criteria. For this, the criteria for updating reviews found in Appendix 4 of the GRADE-ADOLOPMENT paper [11] was used (see Table 3). Based on this information, the Leadership Group made a decision to update the Canadian systematic reviews focusing only on the critical outcomes (see [10] for a list of these for each systematic review) for randomized controlled trials and cohort study designs because the sources of these reviews were older than three months (i.e., they had an end date before November 2016) [11]. We decided not to update the reviews for non-critical outcomes (see [10] for a list of these) or for cross-sectional studies because the consensus was that even if an update was to uncover new studies, they would be graded low quality and as such, would not result in a change to the final guideline. The Australian Leadership Group made the PICOs that guided the four systematic reviews for the 2017 Canadian Guidelines available for comment by the Australian Consensus Panel prior to the Consensus meeting. The Panel was asked to comment on the appropriateness of each of the PICOs for the Australian context. Some of the initial comments sought clarification on the selection of specific search terms for some of the outcomes. These were resolved by indicating that these would be or were captured in the Australian or Canadian searches, respectively, although this information was not clear in the PICOs. Other queries related to the inclusion of information in the summary tables or in the PROSPERO registration or to definitions of specific terms. Where changes were suggested, these were discussed by the Leadership Group and agreement reached. None of the proposed changes were substantial enough to warrant changing any of the existing PICOs.

**Table 3:** Criteria for updating reviews

Criterion	Minor update (all criteria must apply)
Prior Review (for question)	A credible systematic review exists
Full text reviewed for the Research Question of interest	≤20 articles
New Studies	≤5 studies
Evidence profile available?	Available
Outcomes all addressed	All important outcomes addressed

Reference: Appendix 4: GRADE-ADOLOPMENT. [11]

The updates to the four systematic reviews performed for the Canadian Guidelines were conducted with searches completed up to the end of March, 2017. These updated reviews were also fed back to the Canadian Guideline Development Panel to consider as part of their guideline development process. For each systematic review, the quality of evidence was assessed by outcome/indicator and study design, and age group, using the GRADE approach [13, 14]. Each systematic review used the same PICO as the corresponding systematic review completed for the 2017 Canadian Guidelines [15–18].

The results of these systematic review updates were presented at the Consensus Panel meeting from 10 to 12 April 2017. The specific objectives of this meeting were to review, discuss, debate and interpret findings from the Canadian systematic reviews and Australian updated searches, including compositional analyses that were performed on data from Canada, the results of which appear elsewhere in this supplement [19]. Other objectives were to review and adopt/adapt the Preamble and the actual *Canadian 24-Hour Movement Guidelines for the Early Years*; discuss proposed stakeholder consultations; identify research gaps; and plan the launch, dissemination, promotion, integration, and evaluation activities for the *Australian 24-h Movement Guidelines for the Early Years (Birth to 5 years)*.

The process at the Consensus Panel meeting involved reviewing the evidence for each movement behaviour (physical activity, sedentary behaviour, and sleep) individually, starting with the 2017 Canadian systematic reviews and integrating the Australian updates into these reviews. The evidence for each behaviour, including the conclusions of the Canadian review and how this process informed their guidelines was then discussed. The Consensus Panel then followed the GRADE-ADOLOPMENT process to make a decision to adopt or adapt the 2017 Canadian recommendations for each behaviour or create de novo recommendations. In addition, the Panel examined the results of the integrated behaviours systematic review and compositional data analyses from Canada, infused expert opinion into the evidence (such as feasibility, acceptability, equity issues, values and preferences, resources, and balance of benefits and harms), combining evidence of absolute effects across multiple outcomes [20–23], leading to an informed assessment of whether the panel either agreed or disagreed with the judgments made by the Canadian Guideline Development Panel. If the Australian Consensus Panel agreed with the judgments, the recommendations were adopted and the Panel moved on to discuss the guidelines wording. If the Panel disagreed with the judgments, the recommendations were adapted and the Panel moved on to describe the reasons for deviation in the EtD framework. It was noted during the Consensus Panel meeting that a recommendation could be adopted and still added to or translated for adoption in the wording and adjusted if necessary based on this detailed discussion.

The next three sections of the Guideline Development Process [24] are not parts of the GRADE-ADOLOPMENT process but were important components in testing the appropriateness of the adopted guidelines with key stakeholders and in developing plans for the Australian Government (owner of the Guidelines) to consider in the activation of the Guidelines and their potential monitoring and surveillance.

### Stakeholder consultations

The online survey developed as part of the 2017 Canadian Guidelines [10] was modified for the Australian context to seek feedback from stakeholders on their level of agreement with the draft Australian Guidelines emanating from the Consensus Panel meeting. The Human Research Ethics Committee of the University of Wollongong approved administration of the survey and use of a passive consent process (HE 2017/164). The survey sought assessments of the clarity of the title, preamble, and guidelines as well as levels of agreement with the text. Basic demographic information was requested and respondents had an opportunity to provide comments on any aspect of the guidelines. Consensus Panel members were asked to disseminate the survey through their networks, and used a snowball sampling methodology to optimise reach and input from relevant stakeholders. The survey was open from May 18 to June 13, 2017. After the survey closed, numerical responses from participants were tabulated and analysed. Written comments were consolidated into themes and summaries were prepared. The stakeholder survey also allowed respondents to express their interest in publicly disclosing their support for the guidelines pending their review of the final draft. To facilitate this, interested respondents were asked to provide an email address where the final guidelines could be sent.

In addition to the online stakeholder survey, focus groups targeting early childhood educators and parents from different socioeconomic and cultural backgrounds – including Indigenous groups and those who work with children with additional needs – were conducted. These were supplemented with key informant interviews held with a paediatrician; general practitioner with a diploma in Child Health; paediatric physiotherapist; the authority leading the implementation of the National Quality Framework for early childhood education and care services in Australia (ACECQA); and a not-for-profit organization representing long day care owners and operators (Australian Childcare Alliance). These focus groups and interviews sought to understand stakeholders’ perceptions of the *Australian 24-Hour Movement Guidelines for the Early Years*. The focus groups and interviews asked key stakeholders who were difficult to reach through the online survey specific questions about the acceptability and perceived importance, clarity of the guidelines and preamble, facilitators and barriers to implementation and dissemination, and dissemination and implementation recommendations for the Guidelines) [25, 26]. A total of 35 individuals participated in five focus groups (6 participants per focus group) and five interviews (1 participant per interview). Recruitment occurred through existing partnerships and connections. Focus groups and interviews lasted between 30 and 90 mins and were conducted from late-May to mid-September 2017 in the Illawarra region of New South Wales, Sydney, Melbourne, and Perth. The focus groups and interviews were audio-recorded and transcribed verbatim and inductive and thematic data analyses by two researchers were employed and consensus reached on any discrepancies through discussion [27]. Ethics approval was obtained from Human Research Ethics Committee of the University of Wollongong (HE 2017/164). A subcommittee of the Consensus Panel reviewed the survey, focus group and key informant interview results, and suggested revisions to the Guidelines based on the stakeholder feedback, ensuring changes remained true to the available evidence base. Revisions agreed upon by the Leadership Group were then circulated to the entire Consensus Panel for comment and final revisions. Consensus was achieved on the final Guidelines.

### Dissemination, implementation and evaluation plans

A sub-group of the Consensus Panel developed a detailed dissemination, implementation and integration strategy. This included key communication strategies leading up to and beyond the official launch of the Guidelines with government and non-government support for the integration of the guidelines into early childhood education and care settings and into other support services and whole-of-government approaches. Consultation with key users of the guidelines during the Consensus Panel meeting indicated that the most beneficial methods of dissemination, implementation and integration of the guidelines to facilitate behaviour change were: enhancing parent education through the development of an app; using social media to promote actively persuasive messages and materials; training for anticipated end-users (webinars, online, and face-to-face professional development); and a comprehensive social marketing campaign. Expected population health behavioural change and health system impacts and return on investment were modelled based on pilot evidence from previous child and youth guidelines [28].

A phased evaluation plan was also developed. This included initial evaluation of the dissemination and reach of the guidelines and the integrated communications strategy. Ongoing evaluations of community ownership, implementation and integration plan, as well as awareness and knowledge of the guidelines as pathways to long-term improvements in 24-h movement behaviours across early childhood populations were proposed as part of the plan.

### Research gaps and surveillance recommendations

Research gaps were identified through the updates of the systematic reviews and during discussions at the Consensus Panel meeting. This included thinking about how surveillance and monitoring of the new guidelines would occur. The full set of research gaps were distributed to the Consensus Panel after the meeting for further feedback and agreement. A sub-committee met several times via teleconference to make initial recommendations around the monitoring and surveillance of the new *Australian 24-Hour Movement Guidelines for the Early Years* using the same approach as the Canadian Surveillance Sub-committee and having access to the surveillance recommendations table from the Canadian sub-committee [10].

## Results

### Updates to systematic reviews

The full systematic reviews from the 2017 Canadian guidelines are available in this special journal issue [16–18, 29]*.* The results of the updates to the Canadian systematic reviews by the Australian Leadership Group are described below.

For physical activity, 2458 studies were identified from a search of databases, with 24 studies included after screening title and abstracts. Of these, one additional study met the criteria to be included in the update. This was a longitudinal study that assessed physical activity using accelerometry at ages 19 months (*n* = 185) and 42 months (*n* = 116) and motor development at age 5 years [30]. Results showed that moderate- to vigorous-intensity physical activity (MVPA) at 36 months predicted locomotor skills at age 5 years but not object control skills. MVPA at age 19 months was not associated with any motor development outcome at age 5 years. The assessed quality of overall evidence using GRADE criteria for this outcome (“Very Low”) did not change by including this additional study from the updated review.

The sedentary behaviour updated systematic review captured 1820 studies with 99 studies remaining after titles and abstracts were screened. Three additional studies met the inclusion criteria and were included in this update. These comprised one RCT [31] and two longitudinal studies [32, 33]. In the RCT, 70 preschoolers were randomized to watching a fast or slow-paced film to examine the subsequent effect on children’s attention. This was measured by the number of behaviour changes during free-time play. The children who watched the fast-paced film had a higher number of negative behaviour changes compared with those who watched the slow-paced film [31]. The two longitudinal studies comprised 2432 participants aged between 3 years and 5 years at baseline. The first study (*n* = 2411) assessed hours of TV viewing at age 5 and subsequent adiposity and mental health at ages 8, 10, 14, 17, and 20 years [33]. Those children who had lower levels of TV viewing at age 5 had a lower percentage body fat at age 20. There were no associations with mental health. The second study (*n* = 111) used an objective measure of sedentary time and found no association with body composition over a 12mo period among children aged between 3 and 5 years [32]. The assessed GRADE quality of overall evidence did not change for longitudinal studies examining adiposity (“Very Low”) or for RCTs examining psychosocial health (“Moderate”).

For the updated sleep systematic review, 142 studies were identified from the search of databases, with six studies remaining after screening title and abstracts. Three additional studies met the inclusion criteria for the update. These comprised two RCTs [34, 35] (*n* = 108) and one longitudinal study [36] (*n* = 1192) examining the association between sleep duration and cognitive development. The two RCTs found that infants in the nap condition (who were visited after they had a naturally occurring nap) performed better on selected cognitive tasks compared with those in the no-nap condition (who were visited shortly before they were scheduled to take a nap) [34, 35]. The longitudinal study assessed sleep trajectories annually using parent-report from age 2.5 years to 10 years with follow-up at age 10. Results showed that compared to 11-h sleepers, the odds ratio of having poor receptive vocabulary at age 10 was 2.67 [95% confidence interval (CI): 1.24–5.74, *P* = 0.012] for short persistent sleepers and 1.66 (95% CI: 1.06–2.59, *P* = 0.026) for 10-h sleepers [36]. The assessed quality of overall evidence using GRADE criteria for this outcome (“Moderate” for RCTs and “Very Low” for longitudinal studies) did not change as a result of including these additional studies.

The final systematic review update included studies that investigated combinations of physical activity, sedentary behaviour, and sleep and their association with health indicators. The updated searches yielded 518 studies, with five remaining after screening titles and abstracts. No studies met the inclusion criteria and as such, these results were identical to those from the 2017 Canadian review [18].

Agreement in the interpretation of the evidence was reached for each behaviour and for the integration of the three behaviours. Based on the evidence from the Canadian systematic reviews and their GRADE tables and recommendations, in combination with the updated systematic reviews in Australia, the Consensus Panel agreed to adopt the Canadian recommendations. Once it was decided that Australia would adopt the recommendation from the modified EtD framework, the Consensus Panel then decided if they wanted to keep the guideline wording of the Canadian Guidelines. There were a number of minor changes to the wording of the guidelines, preamble and title that were made by the Australian Consensus Panel. Changes were not made to the guideline recommendations per se but rather to the wording of good practice statements [14]. When a change was suggested, the rationale for the change was put forward by the Panel member and discussed. The Panel determined if the proposed change would be consistent with the strength of the evidence recommended and ensured it would not unintentionally alter the interpretation of the guideline. Consensus was required for a change to be accepted. The changes in wording between the Canadian and Australian Guidelines are shown in Table 4. All Consensus Panel members endorsed the draft guidelines and preamble to be used for the stakeholder consultations. As the Canadians were undertaking stakeholder engagement during the period immediately post the Australian Consensus Panel meeting, further minor changes were made to the Canadian Guidelines [10]. Each time this occurred, the Australian Panel was informed and asked if they supported the same change for the Australian Guidelines. In all cases, consensus was reached to either accept or not accept the edit. There were also several instances where changes in wording made to the Australian Guidelines were considered by the Canadian Guideline Development Panel and the same process was followed to determine if this change would be made to the Canadian Guidelines, which it was in several cases.

**Table 4:** Differences in the Australian Guidelines compared to the Canadian Guidelines

Canadian Guidelines (Original)	Australian Guidelines	Reasoning
Title		
***Canadian*** 24-Hour Movement Guidelines for the Early Years (0–4 years): *An Integration of Physical Activity, Sedentary Behaviour and Sleep.*	***Australian*** 24 Hour Movement Guidelines for the Early Years (birth to 5 years): *An Integration of Physical Activity, Sedentary Behaviour, and Sleep.*	To identify the relevant country and age group
Preamble		
These guidelines are relevant to all apparently healthy infants (less than 1 year), toddlers (1–2 years), and preschoolers ***(3–4 years)***, irrespective of gender, cultural background, or the socio-economic status of the family. These guidelines may be appropriate for young children with a disability or medical condition; however, a health professional should be consulted for additional guidance.	These guidelines are relevant to all apparently healthy infants (less than 1 year), toddlers (1–2 years), and preschoolers **(** ***3–5 years)*** **,** irrespective of gender, cultural or ***language background, geographic location***, or socio-economic status of the family. These guidelines may be appropriate for young children with a disability or medical condition; however, a health professional should be consulted for additional guidance.	In Australia, a child must start school before they are aged 6 years old. However some children start aged 4 years old, if they are close to turning 5 years old. Given this variation, the group came to consensus in stating the age group as ***3–5 years.*** This is repeated throughout the Guidelines. Australia is a large country with densely populated capital cities and regional centres. Additionally, there are many rural and remote areas that are geographically isolated due to the Australian climate (wet and dry season in the north of the country). Australia has had a strong migration policy and has attracted a culturally diverse population, especially from Europe and Asia. The Consensus Committee agreed that this wording was more suited to the Australian context.
To encourage healthy growth and development, young children should receive support from ***their*** parents and caregivers that allows for an active lifestyle with a daily balance of physical activities, sedentary behaviours, and sleep. Young children should participate in a range of developmentally appropriate, enjoyable and safe play-based and ***organized*** physical activities in a variety of environments (e.g., home/child care/***school***/community; indoors/outdoors; land/water; summer/winter), both independently as well as interacting with adults and other children. For infants, supervised activities could include tummy time, reaching and grasping, pushing and pulling, and crawling. The quality of sedentary behaviour matters; for example, interactive non-screen based behaviours (e.g., reading, storytelling, singing, puzzles are encouraged. Developing healthy sleep hygiene in the early years is important, this includes having a calming bedtime routine with consistent ***bedtimes*** and wake-up times, avoiding screen time before sleep, and keeping screens out of the bedroom.	To promote healthy growth and development, young children should receive support from parents ***and family***, ***educators*** and caregivers that allows for an active lifestyle with a daily balance of physical activities, sedentary behaviours, and sleep. Young children should participate in a range of developmentally appropriate, enjoyable and safe play-based and ***structured*** physical activities in a variety of environments (e.g., home/***early childhood education and care***/community; indoors/outdoors; land/water; summer/winter), both independently as well as interacting with adults and other children. For infants, supervised activities could include tummy time, reaching and grasping, pushing and pulling, and crawling. The quality of sedentary behaviour matters; for example, interactive non-screen based behaviours (e.g., reading, storytelling, singing, puzzles) are encouraged. Developing healthy sleep hygiene in the early years is important; this includes having a calming bedtime routine with consistent ***sleep*** and wake times, avoiding screen time before sleep, and keeping screens out of the bedroom.	Australia included ***educators*** into this sentence as the Consensus group agreed they were important to identify, separate to caregivers The Australian group agreed on the use of the word ***structured*** in place of organised. ***Early Childhood Education and Care*** is the common terminology used to describe the learning environment of children prior to school entry. Australian group did not use school environment as these children would fall under the Child and Youth Guidelines. Agreed that ***sleep*** time is more appropriate than bed time in that the latter does not indicate the time from which a child actually falls asleep and is inclusive of daytime sleep. This change is repeated throughout the Guidelines. Bedtime in Australia also infers night sleep and we needed to account for full 24-h sleep duration. This is repeated for toddlers and preschoolers.
Guidelines		
**Toddlers (aged 1–2 years)**	**Toddlers (aged 1–2 years)**	
For toddlers, a healthy 24 h includes: • At least 180 min of a variety of physical activities ***at any intensity***, including energetic play, spread throughout the day; more is better;	For toddlers, a healthy 24 h includes: • **Physical activity**: At least 180 min spent in a variety of physical activities including energetic play, spread throughout the day - more is better.	The Australian group chose to utilise sub-headings for the three key areas despite the integrated approach. It was agreed that the use of subheadings assists the reader in understanding the context. These also appear for infants and preschoolers.
• Not being restrained for more than 1 h at a time (e.g., in a stroller or high chair) or sitting for extended periods. For those younger than 2 years, sedentary screen time is not recommended. For those aged 2 years, sedentary screen time should be no more than 1 h; less is better. When sedentary, engaging in pursuits like reading and storytelling with a caregiver is encouraged;	• **Sedentary Behaviour:** Not being restrained for more than 1 h at a time (e.g., in a stroller, ***car seat*** or high chair) or sitting for extended periods. For those younger than 2 years, sedentary screen time is not recommended. For those aged 2 years, sedentary screen time should be no more than 1 h; less is better. When sedentary, engaging in pursuits like reading and storytelling with a caregiver is encouraged.	The Consensus group removed the term ‘at any intensity’ as it was agreed this was redundant given the inclusion of ‘energetic play’.
• 11 to 14 h of good quality sleep, including naps, with consistent bed- and wake-up times.	• **Sleep**: 11 to 14 h of good quality sleep, including naps, with consistent sleep and wake-up times.	The Consensus group agreed to include car seat as one of the examples of equipment where children can be restrained for extended periods. This is repeated in the Infant Guidelines.
Replacing time restrained or sedentary screen time with additional energetic play, and ***trading indoor for outdoor time***, while preserving sufficient sleep, can provide greater health benefits.	For greater health benefits, replace time restrained or sedentary screen time with additional energetic play, while preserving sufficient sleep.	The Australian group agreed we had not assessed evidence to enable consideration of whether or not to include the statement “***trading indoor for outdoor time”.*** Rephrasing of the sentence was also preferred.

The text in bold indicates the differences between the Canadian and Australian Guidelines

### Stakeholder consultations and final guidelines

The draft guidelines developed and approved by the Consensus Panel at their April 2017 meeting were used to seek broader consultation through the online stakeholder survey, focus groups and key informant interviews. At the close of the survey, responses from the 302 participants were tabulated and analysed. The number of responses varied by question with 181 to 249 responses for close-ended questions and 8 to 143 responses for open-ended questions. Respondents were from every state and territory in Australia except Tasmania with 36% from New South Wales, 6.1% from Victoria, 4.4% from Queensland, 28.2% from Western Australia, 5.6% from South Australia, 1.0% from the Australian Capital Territory, and 0.5% from the Northern Territory. Approximately one in five respondents were from outside Australia (18.8%). Respondents identified as being from the following sectors: research/academia (26.7%), early childhood education and care (24.6%), public health (17.1%), healthcare (12.3%), education (8.6%), government (5.4%), sport (2.1%), and physical activity/fitness (1.1%).

The proportion of respondents who strongly agreed or somewhat agreed that all sections of the Guidelines (title, preamble, guidelines) were clearly stated was very high, ranging from 89 to 97%. The proportion who strongly agreed or somewhat agreed with the message in these sections ranged from 91 to 96%. Table 5 provides a detailed breakdown of the responses from the stakeholder survey. Regarding the open-ended questions, the most frequent concerns and suggestions were in relation to identifying the key people or groups for implementing and activating the 24 Hour Guidelines, as well as identifying the support they would require. Changes were made accordingly as described in the Methods section. Many respondents (*n* = 85) indicated interest in publicly supporting the Guidelines once released.

**Table 5:** Summary results of closed-ended stakeholder survey questions

Question	Strongly agree, % (*n*)	Somewhat agree, % (*n*)	Neither agree nor disagree, % (*n*)	Somewhat disagree, % (*n*)	Strongly disagree, % (*n*)	Total responses (*n*)
Is the title clearly stated?	49.8 (124)	39.4 (98)	3.2 (8)	5.62 (14)	2.0 (5)	249
Do you agree with the title?	36.7 (91)	44.4 (110)	10.1 (25)	7.3 (18)	1.6 (4)	248
Is the preamble clearly stated?	60.8 (124)	35.8 (73)	0.5(1)	1.5 (3)	1.5 (3)	204
Do you agree with the preamble?	65.2 (133)	30.8 (63)	2.9 (6)	1.0 (2)	0 (0)	204
The Guidelines are clearly stated	70.9 (139)	25.0 (49)	2.0 (4)	2.0 (4)	0 (0)	196
Do you agree with the Guidelines?	63.3 (124)	27.6 (54)	2.0 (4)	6.1 (12)	1.0 (2)	170
	Much more useful, % (n)	More useful, % (n)	Neutral, % (n)	Less useful, % (n)	Much less useful, % (n)	Total responses (n)
In comparison to separate physical activity,sedentary behaviour, and sleep guidelines, do you find these integrated Guidelines…	42.9 (82)	43.5 (83)	12.6 (24)	0.6 (1)	0.6 (1)	191

Results from the focus groups and key informant interviews supported the findings from the online survey. There was a low awareness of current guidelines but consistent support for the integrated nature of the new Guidelines. Respondents found the Guidelines clear, simple and specific and felt they contained useful and practical information that was perceived, at least by educators, to be already occurring. There was awareness that educators, health workers and parents/carers all play an important role in dissemination and implementation. However, parents felt there needed to be clear messaging to minimise feelings of guilt that may be associated with not meeting the Guidelines. It was also suggested that a glossary of terms be included to provide examples and definitions for some of the terms used in the Guidelines such as sedentary screen time, sleep hygiene, energetic play, and tummy time.

**Fig. 2:**
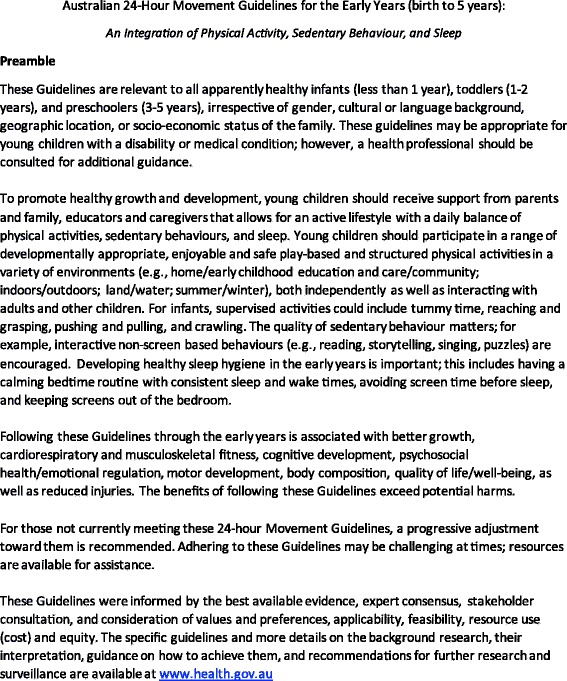
Final Preamble. © 2012–2017 Commonwealth of Australia as represented by the Department of Health; all rights reserved

**Fig. 3:**
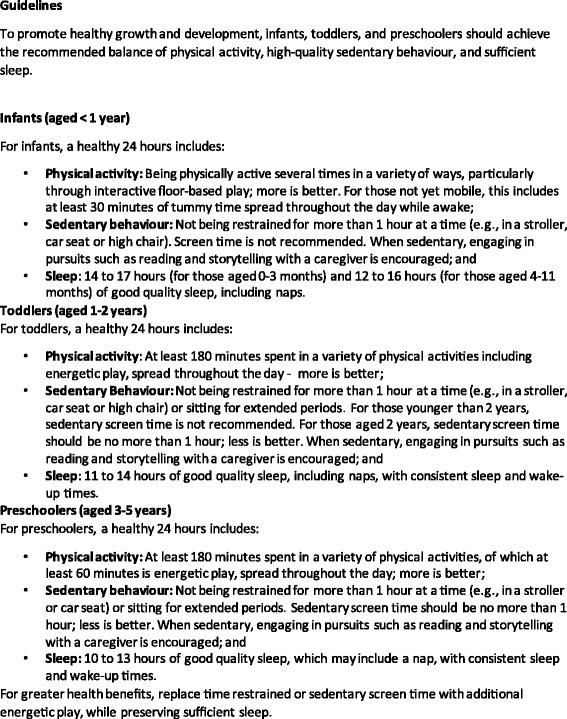
Final Guidelines. © 2012–2017 Commonwealth of Australia as represented by the Department of Health; all rights reserved

A small number of changes were made to the draft Guidelines as a result of the stakeholder consultation. This included the addition of an age range in the title. Around 80% of respondents to the online survey thought the Guidelines should include a specific age range in the title, with nearly 50% of these suggesting they would like this to be expressed as “Birth to 5 years”. Being placed in a car seat was also suggested to be included as an example of restrained sedentary behaviour. In the preamble, it was suggested that the word “family” be included along with “parents, educators, and caregivers” as these are who young children should receive support from to meet the Guidelines. This was based on feedback from the focus groups of the need to be inclusive of all family structures, such as older siblings and grandparents. The final guidelines, including the title and preamble, are provided in Figs. 2 and 3.

### Dissemination, implementation, integration, and evaluation plans

The comprehensive plan to disseminate, implement, integrate and evaluate the guidelines was presented to the Australian Government on 28 June 2017 by a sub-committee of the Leadership Group, including a social marketing expert (Melanie Randle), health economist (SE), and knowledge translation expert (TS). Key aspects of the plan included:

- Coordinating an effective launch of the guidelines and support for guideline dissemination and integration over a three-year period.
- Identifying the health, education, developmental and economic benefits expected with comprehensive dissemination, implementation and integration of the guidelines into early childhood curricula with appropriate community support.
- Assessing the expected multiplier return-on-investment to the health system of investing in well-disseminated and integrated Guidelines for early childhood, given the expected cost savings from improving the trajectory of integrated movement behaviours and lifestyles from early childhood.
- Ensuring maximum reach and dissemination of the guidelines and making them part of public culture.
- Identifying target audiences and how to reach and engage them.
- The planning and development required to inform social marketing and creative idea development and refinement to actively persuade uptake and reduce perceived costs of improving movement behaviours. This has been identified [28] as key in optimising community ownership of key messages, parent, practitioner and child choices, and long term behaviour change.
- Describing the web-based “digital hub”, stakeholder outreach, and comprehensive communications strategies needed to facilitate sustained implementation and activation of the guidelines following the initial guideline launch, including a social media strategy.
- Specifying components of the post-launch campaign for parents/carers and educators (primary target audience) and other key influencers.
- Describing the resources required for a comprehensive approach to optimising guideline impacts and their expected cost.
- Evaluating changes in awareness and knowledge of the guidelines and in child movement behaviours.

### Research gaps and surveillance recommendations

Research gaps were identified through the updated systematic reviews and during discussion at the Consensus Panel meeting and are summarised in Table 6. These were determined independent of the research gaps identified in the Canadian Guideline development process. As a result, there may be some overlap between the two countries. As 24-h movement guidelines are new in the early years, there are many gaps in the research and evaluation, providing fertile ground for researchers in the future.

**Table 6:** Summary of research needs to address gaps in relation to the development of 24-h integrated movement guidelines for the early years

Research needs
General
• Timing and consistency studies needed for sedentary behaviour, physical activity and sleep
Physical activity
• More accurate ways of objectively measuring physical activity are needed (currently no valid and reliable accelerometer cut points for infants).
• More research needed to determine how MVPA is defined for young children, given the sporadic nature of their activity.
• More evidence on the associations between light-intensity physical activity and health and development outcomes is needed and how light-intensity physical activity is defined.
• Better evidence needed for “a variety of ways” (for infant guidelines).
• More evidence needed overall for infants.
Sedentary behaviour
• No evidence was “high quality” (only 2 RCTs and several limitations across studies).
• Only one longitudinal study used objective measures of sedentary behaviour (e.g., accelerometers).
• No studies examined newer/evolving technologies that contribute to sedentary time (e.g., tablets, FaceTime/Skype, small screens); only 1 study examined mobile phone use.
• Few studies examined certain sedentary behaviour exposures (e.g., sitting, supine position, reading, internet, sedentary quiet play).
• Difficult to define and measure “sedentary behaviour” in infants given the child/adult concept of “breaking up sedentary behaviour” may not be relevant to non-walking infants.
Sleep
The review only focused on sleep duration
• Many other important factors beyond sleep duration should be considered in the development of sleep recommendations, including aspects of sleep *quality* such as sleep efficiency (i.e., proportion of the sleep opportunity spent in sleep), timing (i.e., bedtime/wake-up time and naps), sleep architecture (i.e., the number of different sleep stages and composition of sleep in general), consistency (i.e., day-to-day variability, seasonal changes), and sleep consolidation (i.e, organization of sleep across the night, amount of waking after sleep onset, etc)
• In addition, sleep duration in the early years is generally comprised of both daytime and night time sleep. However, it has been reported that the effects of daytime sleep on health may not be the same as night time sleep, with positive effects of sleep duration suggested to relate to the stage in sleep transition from polyphasic to monophasic sleep during which naps cease Multiple age groups (e.g., toddlers, preschoolers) were also grouped together, despite obvious differences in development
• Development progresses rapidly during the early years and many factors could have confounded the associations that have been reported (e.g., growth, eating habits, environment, locomotion, etc.)
• Ideally, future research should use narrower age groups that are aligned with the current sleep duration recommendations (e.g., newborns [0–3 months], infants [4–11 months], toddlers [1–2 y], preschoolers [3–5 y])
The available evidence relies heavily on cross-sectional studies that use parent-reported sleep durations
• Subjective sleep reports are less reliable than objective measures of sleep. It is also well-known that parent-reported sleep duration overestimates actual sleep duration compared with objective measures.
• Subjective sleep reports are therefore valid for screening, but are less consistent and reliable in estimating sleep pattern variables such as sleep duration, night wakings, and sleep onset latency (Bauer and Blunden, 2008).
• Even when objective measurements are used, there is a wide variety of largely incommensurable metrics for duration, efficiency and fragmentation of sleep. Where possible, future research should include objective measures of sleep, with agreed metrics. Additionally, where only subjective measures are included the questions used to evaluate sleep should be carefully selected as this can greatly impact the validity of self-report

A sub-committee examined the surveillance recommendations made by the Canadian Guideline Development Panel (see [10]) and considered these for adoption in the Australian context. The Australian sub-committee adopted the Canadian recommendations and agreed with the rationale for those guidelines which were not recommended for surveillance until further research has been completed (see Table 7).

**Table 7:** Surveillance recommendations for the Australian 24-Hour Movement Guidelines for the Early Years (birth to 5 years). (adapted from the Surveillance recommendations for the Canadian 24-Hour Movement Guidelines for the Early Years)

Physical activity			
Australian Guideline	Specific Surveillance Recommendation	Rationale for specific surveillance recommendation	Recommendation for minimum inclusion in overall guideline surveillance^a^
Infants (aged <1 year)			
Being physically active several times in a variety of ways, particularly through interactive floor-based play; more is better.	None	Currently there are no available benchmarks, further research is required	No
For those not yet mobile, this includes at least 30 min of tummy time spread throughout the day while awake	Total tummy time on the previous day is ≥30 min while awake^b^	A representative day provides a more accurate recall and hence better estimate of the prevalence of the guideline in a population representative sample [37, 38].	Yes
Toddlers (aged 1–2 years)			
At least 180 min spent in a variety of physical activities at any intensity, spread throughout the day; more is better.	Previous day physical activity is ≥180 min with at least some energetic play (MVPA)^b^	A representative day provides a more accurate recall and hence better estimate of the prevalence of the guideline in a population representative sample [37, 38]. It allows direct comparison with previous national representative data from the Australian Health Survey [39]	Yes
Including energetic play	Previous day total physical activity is ≥180 min with at least some energetic play (MVPA)^b^	As there are no benchmarks for duration we suggest not having a minimum threshold for energetic play or MVPA in this age group.	No
Preschoolers (aged 3–5 years)			
At least 180 min spent in a variety of physical activities spread throughout the day	Previous day total physical activity is ≥180 minutes^b^	A representative day provides a more accurate recall and hence better estimate of the prevalence of the guideline in a population representative sample [37, 38]. It allows direct comparison with previous national representative data from the Australian Health Survey [39]	Yes
Of which at least 60 min is energetic play; more is better	Previous day MVPA is ≥60 minutes^b^		Yes
Sedentary behaviour			
Guideline	Specific Surveillance Recommendation	Rationale for specific surveillance recommendation	Recommendation for minimum inclusion in overall guideline surveillance
Infants (aged <1 year)			
Infants			
Not being restrained for more than 1 h at a time (e.g., in a stroller, car seat or high chair).	Time spent restrained is ≤1 h at a time^d^	Empirical evidence substantiating this threshold is lacking though this threshold is aligned with earlier guidelines and has met with stakeholder and end-user acceptance	No
When sedentary, engaging in pursuits such as reading and storytelling with a caregiver is encouraged	None	Currently there are no available benchmarks, further research is required.	No
Toddlers (aged 1–2 years)			
Not being restrained for more than 1 h at a time (e.g., in a stroller, car seat or high chair).	Time spent restrained is ≤1 h at a time^d^	Empirical evidence substantiating this threshold is lacking though this threshold is aligned with earlier guidelines and has met with stakeholder and end-user acceptance	No
Or sitting for extended periods	None	Currently there are no available benchmarks to be more specific for “sitting for extended periods”, further research is required.	No
When sedentary, engaging in pursuits such as reading and storytelling with a caregiver is encouraged	None	Currently there are no available benchmarks, further research is required	
Preschoolers (aged 3–5 years)			
Not being restrained for more than 1 h at a time (e.g., in a stroller or car seat).	Time spent restrained is ≤1 h at a time^d^	Empirical evidence substantiating this threshold is lacking though this threshold is aligned with earlier guidelines and has met with stakeholder and end-user acceptance	No
Or sitting for extended periods	Bouts of sedentary time	Currently there are no available benchmarks to be more specific for “sitting for extended periods”, further research is required.	No
When sedentary, engaging in pursuits such as reading and storytelling with a caregiver is encouraged	None	Currently there are no available benchmarks, further research is required	No
Screen time			
Guideline	Specific Surveillance Recommendation	Rationale for specific surveillance recommendation	Recommendation for minimum inclusion in overall guideline surveillance
Infants (aged <1 year)			
Screen time is not recommended.	Previous day includes no screen time^c^	A representative day provides a more accurate recall and hence better estimate of the prevalence of the guideline in a population representative sample [37, 38]. This threshold is aligned with earlier guidelines and has met with stakeholder and end-user acceptance, and is consistent with evidence in this age group indicating that no screen time is better than some screen time and that less screen time is better than more screen time, for health and development.	Yes
Toddlers (aged 1–2 years)			
For those younger than 2 years, sedentary screen time is not recommended.	Previous day includes no screen time^c^	A representative day provides a more accurate recall and hence better estimate of the prevalence of the guideline in a population representative sample [37, 38].	Yes
For those aged 2 years, sedentary screen time should be no more than 1 h per day; less is better	Sedentary screen time on previous day is ≤1 hour^b^	A representative day provides a more accurate recall and hence better estimate of the prevalence of the guideline in a population representative sample [37, 38]. It allows direct comparison with previous national representative data from the Australian Health Survey [39]	Yes
Preschoolers (aged 3–5 years)			
Sedentary screen time should be no more than 1 hour per day; less is better.	Sedentary screen time on previous day is ≤1 hour^b^	A representative day provides a more accurate recall and hence better estimate of the prevalence of the guideline in a population representative sample [37, 38]. It allows direct comparison with previous national representative data from the Australian Health Survey [39]	Yes
Sleep			
Guideline	Specific Surveillance Recommendation	Rationale for specific surveillance recommendation	Recommendation for minimum inclusion in overall guideline surveillance
Infants (aged <1 year)			
14 to 17 h (for those aged 0–3 months) of good quality sleep, including naps.	Sleep period time on previous night (offset minus onset), plus daytime naps for previous day.	Currently recommended by NSF, based on expert opinion. https://sleepfoundation.org/press-release/national-sleep-foundation-recommends-new-sleep-times/page/0/1	Yes
12 to 16 h (for those aged 4–11 months) of good quality sleep, including naps.	Sleep period time on previous night (offset minus onset), plus daytime naps for previous day.	Currently recommended by NSF^e^, based on expert opinion. https://sleepfoundation.org/press-release/national-sleep-foundation-recommends-new-sleep-times/page/0/1	Yes
Toddlers (aged 1–2 years)			
11 to 14 h of good quality sleep, including naps,	Sleep period time on previous night (offset minus onset), plus daytime naps for previous day.	Currently recommended by NSF, based on expert opinion. https://sleepfoundation.org/press-release/national-sleep-foundation-recommends-new-sleep-times/page/0/1	Yes
With consistent sleep and wake-up times	Day-to-day variability in sleep onset and offset times.^f^	Recommended as part of sleep hygiene. No evidence in this age group, but there is some evidence in adolescents (https://www.ncbi.nlm.nih.gov/pubmed/28129442) and adults (https://www.ncbi.nlm.nih.gov/pubmed/27091639)	No
Preschoolers (aged 3–5 years)			
10 to 13 h of good quality sleep, which may include a nap,	Sleep period time on previous night (offset minus onset), plus daytime naps for previous day.	Currently recommended by NSF, based on expert opinion. https://sleepfoundation.org/press-release/national-sleep-foundation-recommends-new-sleep-times/page/0/1	Yes
With consistent sleep and wake-up times	Day-to-day variability in sleep onset and offset times.^f^ Bedtime and wake-up time should not typically vary by more than ±30 min including on weekends^g^	Recommended as part of sleep hygiene. No evidence in this age group, but there is some evidence in adolescents (https://www.ncbi.nlm.nih.gov/pubmed/28129442) and adults (https://www.ncbi.nlm.nih.gov/pubmed/27091639)	No

^a^These indicate the current recommended minimum inclusion recommendations for surveillance of meeting the 24-h guidelines. Other specific guideline recommendations, which have not been identified as recommended components for surveillance of meeting the 24-h guidelines, should still be measured for descriptive purposes and to determine if changes are occurring prospectively. As evidence grows and surveillance measures evolve for these other recommendations, updates to the minimum surveillance criteria may be required

^b^If multiple representative day recalls are available (e.g., last three days) it is recommended to use these over just the previous day

^c^It is understood that under special circumstances exposure to screen time may happen but should be rare or unusual

^d^It is understood that under special circumstances being restrained in excess of 1 h at a time may occur but should be rare or unusual

^e^Note that the NSF actually recommends 12–15 h for this age group

^f^Surveillance requires the use of sleep diaries recording onset and offset times. Until there are better dose-response relationships between sleep variability and health outcomes, it is premature to recommend a particular goal (e.g. “within 30 min”). There are many ways this can be quantified. The simplest is the SD of onset and offset times over one week

^g^To accurately assess consistency of bedtime and wake-up time data should be collected on both weekday and weekend days. If data from weekday and weekend days are available, it is recommended that the average variation in bedtime and wake-up time be weighted 2/7 for weekend days and 5/7 for weekdays to most accurately reflect average weekly measures

The Australian sub-committee recommended using a representative day (e.g., previous day) for surveillance of each of the behaviours rather than an average day (as recommended by the Canadian Surveillance Sub-committee). The rationale for recommending a representative day was that it would provide a more accurate recall and hence better estimate the prevalence of the guideline in a population representative sample [37, 38]. It would also allow direct comparison with previous national representative data collected using the same approach as part of the Australian Health Survey [39].

## Discussion

This paper describes the process and outcomes to develop the *Australian 24-Hour Movement Guidelines for the Early Years (Birth to 5 years): An Integration of Physical Activity, Sedentary Behaviour, and Sleep*. These integrated guidelines represent a shift in thinking away from separate guidelines for each of these behaviours. The feedback to date is that this integrated approach has been well received by key stakeholders. The Australian Consensus Panel was also positive in their response to the task of developing integrated guidelines. This was made considerably easier by having the draft Canadian guidelines to refer to and that some panel members were experienced with the 24-h approach to guideline development. We believe our guideline development process was comprehensive, transparent and rigorous. A strength of the Guidelines is the diversity among the leadership group (both in being multidisciplinary and including researchers and representatives from government and non-government organisations) who provided advice on all aspects of the process. A new feature in Australia movement guideline development was the inclusion of a GRADE methodology expert on the panel (DGh) who was especially helpful in ensuring the panel followed the GRADE process and advised on the ADOLOPMENT approach. The composition of the Consensus Panel was also diverse and included researchers from across the movement continuum (sleep, sedentary behaviour, and physical activity), clinicians, policy making, evidence synthesis and health economics experts, and key stakeholders from the early childhood education and care sector and parent organisations. Involvement of international experts (Canada and New Zealand) provided an opportunity to harmonize guidelines across countries.

Australia used a hybrid process to develop the Australian Guidelines. It involved adopting the Canadian Guidelines using a method firmly grounded in GRADE and based on the GRADE-ADOLOPMENT approach [9]. On the basis of following this process, the judgments of the Australian Consensus Panel did not differ sufficiently to change the directions or strength of the recommendations and as such, the Canadian recommendations were adopted. The advantages of this included being able to extend the Canadian guideline development work to the Australian context and thus develop guidelines in a much shorter space of time and at a substantially reduced cost. As such, we would recommend the GRADE-ADOLOPMENT approach, especially if a credible set of guidelines and related materials (e.g., PICOs) and a transparent process are available to be scrutinized. We were fortunate that our Canadian colleagues were willing to share this information prior to it being published. This avoided what would otherwise have been considerable duplication of efforts if Australia were required to undertake de novo guideline development. In turn, the Australian Leadership Group were able to feed back to the Canadian Guideline Development Panel the results from our updated reviews and any changes to the wording of the Guidelines, highlighting the reciprocal benefits of our approach. It is feasible that other countries may consider using this approach when developing and or revising national movement behaviour guidelines.

While it was beneficial for Australia to have access to the recently completed Canadian systematic reviews and GRADE tables, and the ADOLOPMENT approach to follow, this process has not been without its challenges. The most notable being that we were unable to follow every step of the GRADE-ADOLOPMENT process due to the Canadian GRADE process not being fully completed. For example, it was not possible to follow Steps 4 or 5 (credibility assessment and recording of details about the guidelines and evidence synthesis) because the Canadian guidelines had not yet been published. This meant that there was some information about EtD criteria available, but it was not completed (Step 5 of Fig. 1). As such, we were able to only use part of an EtD framework. From a methodological perspective, it would be preferable to have had a longer time period so that a complete GRADE EtD framework could have been used. However, the contract with the Australian Government stipulated that the final guidelines needed to be developed and submitted by 30 June 2017 meaning it was not possible for the Australian Guidelines Consensus Panel to wait until all Canadian information was available allowing the full GRADE-ADOLOPMENT process to followed.

While we ended up having the same PICO elements as those in Canada, it would have been a challenge to the ADOLOPMENT process if we wanted to change these, for example, to add an outcome. While this would have been quite innocuous if developing guidelines de novo, it would have meant re-running systematic reviews and this would have been a challenge given the timeline. The Leadership Group also discussed whether to include cross-sectional studies in the updated systematic reviews. It was decided that even if several studies were found, the level of evidence would unlikely be enough to change the overall recommendation. As it was highly likely studies using these designs would not make a difference the Leadership Group decided not to change the PICO. However, it is important to note that this decision was somewhat influenced by the limited timeframe and resources for the Australian guideline development. In future, it would be optimal if there was an initial face-to-face meeting of the Consensus Panel to discuss the Canadian PICOs and their appropriateness for the Australian context rather than trying to do this over email. Due to the size of the Consensus Panel, the project budget, and the short timeline, it was not feasible for a face-to-face meeting to be held for this task.

The Guidelines, while integrating all movement behaviours, remain consistent with the current *National Physical Activity Recommendations for Children Aged 0–5* [1]. There is the inclusion of a preamble, which is designed to provide context for the guidelines and include good practice statements. While these statements are not all evidence-based, they provide context and serve to advise those for whom the guidelines are relevant, of the types of activities that parents/carers can adopt to help children meet the guidelines, the benefits of doing so, links to helpful resources, and a statement on how the guidelines were developed. The major change to the existing guidelines is the integration of all movement behaviours across a 24-h period, with the most notable additions being specific recommendations regarding energetic play and sleep duration. There is growing evidence that moderate- to vigorous-intensity physical activity (MVPA, operationalised as energetic play for the guidelines) is associated with greater health benefits than activity that is less intense. This evidence was not available when the initial Australian guidelines were developed. The amount of tummy time for infants has also been quantified (30 mins/day) based on additional evidence about the beneficial duration of this behaviour.

Although presented as “24-h movement guidelines”, they are not prescriptive recommendations that add exactly to 24 h, (e.g., for preschoolers, at least 3 h of physical activity, no more than 1 h of sedentary screen time or less than 1 h of being restrained, and 10–13 h of sleep). For example, if one child sleeps 13 h and another 10 h, the latter has three additional hours of time to be distributed among the wake-time behaviours. In addition, some degree of day-to-day variability, such as that across a week given different activities on different days, is normal, and provision of ranges allows for this flexibility. For these reasons, and to be accommodating to different schedules and changes in schedules, the guidelines provide recommendations such as “replace time restrained or sedentary screen time with additional energetic play” and “not sitting for extended periods” to give directional advice while recognising the dynamics of the component behaviours between and within individuals. Collectively, guidance for healthy movement behaviours over the whole day is provided.

### Release, dissemination, implementation, integration, and evaluation planning

The new guidelines were co-released with Canada on November 21, 2017. Supporting the release by the Australian Government Health Minister were a social media strategy, and web resources housed on the Australian Department of Health website. Professional development for educators and other professionals delivered by ACECQA and state health departments will support their training post-release. At a minimum, the impact and success of the launch of the new guidelines will be assessed using indicators of dissemination reach. A plan has been developed for a comprehensive evaluation of the subsequent dissemination, implementation, and integration activities to assess community ownership and population-level community impacts on early childhood movement behaviours over time.

### Updating the guidelines

The final stage in the guideline development process is the planning of updates and revisions [24]. The Australian Consensus Panel recommends that these guidelines be reviewed, and updated if necessary, at least every 10 years or when significant new research emerges warranting change.

### Strengths and limitations

A strength of our hybrid guideline development approach was the prospective collaboration that resulted in two sets of National Guidelines being developed independently while using the same body of evidence. It allowed some cross-pollination and provided governmental co-operation between countries to enable co-release of the Guidelines.

A limitation of the Australian Guidelines is that they are for ages birth to 5 years, however, the systematic reviews from Canada upon which the evidence was based, covered the ages birth to 4 years. This reflected differences in schooling systems in each country. It was the opinion of the Consensus Panel that it would be highly unlikely that we would have missed any studies among 5 year-olds that that would have changed the strength of the recommendation and we are confident that the same recommendations would apply. Most research on which this is based is on children before they reach school – this structural distinction of school versus not school is more important for the guidelines than the biological age of the children.

Because of the recent development of the ADOLOPMENT approach, there was some uncertainty among the leadership group as to whether it was being followed correctly. While we do believe there is merit in the approach, we would make the following suggestions:

1. Great clarity on the correct stages of the process. For example, Fig. 1 in the Schünemann et al. paper [11], provides a description of the steps to follow. Indicating how this Figure then relates to Appendices 3 and 4 and the Steps outlined in Appendix 1 could be described.
2. Ensure there is a communication channel between those seeking to follow the GRADE-ADOLOPMENT process and those whose guidelines they are seeking to adopt or adapt. A secondary suggestion would be for guideline developers to be transparent in their development approach and to publish or make publicly available all aspects of their development process to facilitate ADOLOPMENT efforts.
3. In undertaking an assessment of strength of evidence, GRADE-ADOLOPMENT rules (e.g. strong evidence with 60% of trials statistically significant) could be improved to better inform guidelines/decision-making with best practice consideration of strength of cumulative evidence. For example 59/100 trials with statistically significant positive findings may be considered stronger than 3/5 trials.
4. In applying evidence synthesis to health promotion settings such as that underlying integrated movement guidelines [5], consideration should be given to evidence triangulation across different evidence types. In particular triangulating pre-post and case-control analysis where key weaknesses of each approach independently can be addressed with triangulated evidence [40, 41].
5. The Leadership Group and Consensus Panel are given training in the ADOLOPMENT approach prior to using and implementing it.
6. Ensuring that there is a nominated person in the group who is responsible for ensuring that all steps in the ADOLOPMENT process are being followed correctly (e.g., an ADOLOPMENT Advisor).

## Conclusion

To our knowledge, this is the first time the GRADE-ADOLOPMENT approach has been used. On the basis of following this approach, the judgments of the Australian Consensus Panel did not differ sufficiently to change the directions and strength of the recommendations and as such, the Canadian recommendations were adopted. The new *Australian 24-Hour Movement Guidelines for the Early Years (Birth to 5 years): An Integration of Physical Activity, Sedentary Behaviour, and Sleep* represents a new way of thinking about daily movement behaviours among our young children. It focuses on the integration of movement across a whole day and the way changes in one behaviour may impact another. The evidence we reviewed supports promotion of healthy growth and development in the early years, through a balance of the recommended sleep, physical activity and high-quality sedentary behaviours such as reading, singing, and playing with toys with a parent/carer every day. Adherence to these recommendations is associated with enhanced health and development in young children and exceeds potential harms. These guidelines are relevant to all apparently healthy infants, toddlers and preschoolers. It is hoped that developing healthy physical activity, sedentary, and sleep behaviours in the first five years of life will establish a positive health trajectory that can be sustained across the life course.

## Abbreviations

ACECQA: Australian Children’s Education & Care Quality Authority
ADOLOPMENT: A methodology that combines the advantages of adoption, adaptation, and de novo development of recommendations based on the GRADE EtD frameworks
AGREE: Appraisal of Guidelines for REsearch & Evaluation
ARC: Australian Research Council
ECA: Early Childhood Australia
EtD: Evidence-to-decision frameworks
GRADE: Grading of Recommendations Assessment, Development, and Evaluation
MVPA: Moderate- to Vigorous-intensity Physical Activity
NHMRC: National Health & Medical Research Council
NSW: New South Wales
PICO: Population, Intervention, Comparator, and Outcome
PROSPERO: The Prospective Register of Systematic Reviews
UOW: University of Wollongong
